# Androgen deprivation therapy does not increase rates for reintervention, complication, or infection in primary penile implant or artificial urinary sphincter surgery: a retrospective cohort study from the TriNetX network

**DOI:** 10.1038/s41443-025-01015-8

**Published:** 2025-01-29

**Authors:** Zachary J. Prebay, David Fu, Aaron R. Hochberg, Paul H. Chung

**Affiliations:** Department of Urology, Sidney Kimmel Medical College at Thomas Jefferson University, Philadelphia, PA USA

**Keywords:** Urethra, Erectile dysfunction, Urinary incontinence, Risk factors, Adverse effects

## Abstract

Prostate cancer treatment-related erectile dysfunction and stress urinary incontinence are commonly treated with inflatable penile prosthesis (IPP) or artificial urinary sphincter (AUS). Given the association with androgens and penile/urethral health, we aim to evaluate whether patients on androgen deprivation therapy (ADT) undergoing IPP or AUS surgery are at increased risk for reintervention, complication, or infection. We queried the TriNetX database for adult males receiving IPP or AUS. The ADT cohort included those on ADT 3 months before or any time after surgery. We performed sub-analysis for leuprolide and bicalutamide. Cohorts and outcomes were defined by Current Procedural Terminology and International Classification of Diseases codes. Propensity score matching was performed using age, prostate cancer, history of prostatectomy, and history of radiation. Outcomes were reintervention (revision, removal, or replacement), infection, and complication. Analytics were performed in March 2024. 13,432 patients received an IPP and 5676 received an AUS, 465 and 745 of whom were on ADT, respectively. The only significant AUS analysis was for patients on abiraterone having fewer reinterventions (10.5% vs 20.8%, RR = 0.50 [0.29, 0.88]). Patients receiving an IPP with ADT had fewer reinterventions (7.2% vs 12%, RR = 0.60 [0.39, 0.92]) and complications (12.7% vs 18.5%, RR = 0.68 [0.49, 0.95]). Those on a GnRH agonist had fewer reinterventions (7.4% vs 11.7%, RR = 0.63 [0.41, 0.98]) for IPP. Patients receiving an IPP on bicalutamide had fewer reinterventions ( <5.2%* vs 10.8%, RR = 0.48 [0.23, 0.99]) and on leuprolide had fewer complications (12.2% vs 19.3%, RR = 0.63 [0.43, 0.91]). The remainder of analyses showed no significant differences. Patients with IPP or AUS do not fare worse on ADT. Further evaluation into the duration of ADT may provide clinical context, but based on these results, ADT should not limit implant surgery.

## Introduction

Prostate cancer treatment incurs notable side effects, namely erectile dysfunction (ED) and stress urinary incontinence (SUI) [1, 2]. Both conditions can significantly reducte quality of life [3]. Once conservative treatment measures have failed, corrective options include placement of genitourinary implants, such as inflatable penile prosthesis (IPP) for ED and artificial urinary sphincter (AUS) for SUI.

With cancer progression, a patient may require treatment with androgen deprivation therapy (ADT) [4, 5] which aims to block growth of the androgen-dependent malignant prostatic cells. While effective, ADT carries potential concerns such as diminished bone health, metabolic dysregulation, and accelerated cardiovascular disease [5, 6]. Androgens have also been implicated in processes related to penile and urethral tissue health. Several animal and human studies have shown that castration results in decreased androgen receptor expression in spongiosal tissue and reduced periurethral vascularity [7–9]. Clinically, urologic implant outcomes have been found to be negatively impacted by hypogonadism. Previously, our group demonstrated that hypogonadal patients undergo more reinterventions and experience more complications and infections following AUS or IPP implant surgery [10]. While there is an incomplete understanding for why hypogonadism impacts urologic implant outcomes, it seems that a relationship exists.

It is unknown whether patients on ADT experience similar outcomes as those with physiologic hypogonadism. We set out to explore this possibility, as reoperations and complications can be costly and detrimental to patients [11–13]. Some patients may not even be offered an implant due to the potential concerns for adverse outcomes in the setting of ADT. Our objective was to utilize a large, multi-national research database to investigate whether patients on ADT experience different outcomes following AUS and IPP placement. We hypothesize that patients on ADT will display increased instances of reinterventions, device complications, and infections following surgery.

## Materials and methods

### Data source

Data were collected from the TriNetX Global Collaborative Network (www.trinetx.com). TriNetX is a large, multi-national database containing historical electronic health record (EHR) data from over 140 million patients across 115 healthcare organizations. The database continually updates, but only reports data coded into a patient’s EHR from up to 20 years prior to the date of analysis (2004–2024), excluding those undergoing the index event prior to this date. The database was queried, and cohorts were established using Current Procedural Terminology (CPT), International Classification of Diseases-10 (ICD), and TriNetX curated medication codes (which utilizes Veterans Affair Drug Classification and RxNorm Concept Unique Identifiers). TriNetX maps ICD-9 codes to ICD-10 for ease of analysis.

### Study population

TriNetX was queried for all adult males undergoing AUS (CPT 53445) and IPP (CPT 54401 or 54405) implant surgery, which was defined as the index event. ADT status was defined as being on an anti-androgen medication anytime from 3 months prior to index event to any time after. The following ADT medications were included: leuprolide, bicalutamide, flutamide, nilutamide, apalutamide, darolutamide, relugolix, degarelix, and abiraterone. We did not evaluate duration of ADT.

### Outcomes and statistical analysis

The primary outcome was rate of reintervention, defined as any repair (CPT 53449, 54408), removal (CPT 53446, 54406/54415), replacement (CPT 53447, 54410/54416), or replacement through an infected field (CPT 53448, 54411/54417) procedure for both AUS and IPP, respectively. Insertion of a semi-rigid penile prosthesis (CPT 54400) was also considered a reintervention for IPP patients. Secondary outcomes were rate of complication (ICD T83) and rate of infection (T83.5 or T83.6). The primary comparison was patients on ADT to patients not on ADT. Sub-analyses included all patients on a single medication type (i.e. GnRH agonists, GnRH antagonists, abiraterone) against patients not on ADT. We also compared patients on leuprolide and bicalutamide, respectively, to patients not on ADT as these were the two most prescribed ADT medications in our cohort.

Propensity score matching (PSM) was performed for cohorts in all comparisons to limit potential confounding factors. TriNetX provides a built-in feature for PSM with specified variables of interest, utilizing 1:1 greedy-nearest neighbor matching to generate equal sized cohorts. The following variables were utilized for PSM: age, history of prostate cancer (ICD C61), history of prostatectomy (CPT 55840, 55842, 55845, 1014183), and history of radiation therapy (Z92.3). Demographic data for the AUS and IPP cohorts were collected. All analyses were done directly on the TriNetX website and were run in March 2024. Analysis included descriptive statistics to characterize cohorts and calculation of risk ratios (RR) and 95% confidence intervals (CI) to determine differences between cohorts. For reintervention in our primary analysis, we also generated Kaplan-Meier Curves and report median survival, log-rank test, and hazard ratios (HR) with 95% CI. Of note, for analyses that result in less than or equal to 10 patients, TriNetX rounds the value to 10 as a part of a patient privacy protection mechanism. Data affected by this mechanism are denoted by an asterisk (*).

### Ethical approval

Regarding ethical approval, per the TriNetX publication guidelines “This retrospective study is exempt from informed consent. The data reviewed is a secondary analysis of existing data, does not involve intervention or interaction with human subjects, and is de-identified per the de-identification standard defined in Section §164.514(a) of the HIPAA Privacy Rule. The process by which the data is de-identified is attested to through a formal determination by a qualified expert as defined in Section §164.514(b)(1) of the HIPAA Privacy Rule. This formal determination by a qualified expert refreshed on December 2020.” (https://trinetx.com/real-world-resources/case-studies-publications/trinetx-publication-guidelines/, accessed January 12, 2025).

## Results

### AUS patients

5676 patients were identified as having received an AUS. From this group, 743 (13.1%) were on ADT and 4933 (86.9%) were not on ADT during the specified time period (Table 1). PSM resulted in cohorts of 743 patients each with similar variables of interest (Supplementary Table 1A). Of patients on ADT, 693 were on GnRH agonists, 56 were on GnRH antagonists, and 180 were on abiraterone after PSM (Supplemental Table 1B–D). Patients on ADT experienced similar rates of reinterventions (19.4% vs 23.0%, RR = 0.85 [0.69, 1.0]), complications (22.0% vs 24.5%, RR = 0.90 [0.73, 1.1]), and infections (5.5% vs 7.6%, RR = 0.72 [0.49, 1.1]) compared to patients not on ADT (Table 2). On Kaplan Meier analysis, median survival was only met for reinterventions, where patients on ADT showed shorter median survival (4007 vs 5627 days), but with no significance on log-rank test or hazard ratio (Supplemental Fig. 1).

**Table 1:** Baseline demographics – artificial urinary sphincter.

	Artificial Urinary Sphincter (Total) N = 5676	Artificial Urinary Sphincter (On ADT) N = 743	Artificial Urinary Sphincter (Not On ADT) N = 4933
**Age (y)**	69.3 +/− 9.3	70.3 +/− 7.8	69.3 +/− 9.5
**Race**			
Asian	2%	<1%*	2%
American Indian or Alaska Native	<1%	0%	<1%*
Black or African American	11%	10%	12%
Native Hawaiian or Other Pacific Islander	1%	0%	<1%*
White	74%	80%	74%
Other Race	3%	3%	3%
Unknown Race	9%	12%	9%
**Prostate cancer (ICD C61)**	68%	94%	65%
**Prostatectomy**			
(CPT 55840)	1%	1%*	1%
(CPT 55842)	<1%	0%	<1%
(CPT 55845)	3%	4%	3%
(CPT 1014183)	4%	6%	4%
**Personal history of irradiation (ICD Z92.3)**	11%	30%	10%

*ADT* androgen deprivation therapy.

*Denotes ≤10 instances.

**Table 2:** Risk of reintervention, complication, and infection for patients receiving artificial urinary sphincter.

Comparison	N before PSM	N after PSM	Reintervention (%, Risk Ratio [95% CI])	Complication (%, Risk Ratio [95% CI])	Infection (%, Risk Ratio [95% CI])
On ADT vs Not On ADT	743 4933	743 743	19.4% 23.0% 0.85 [0.69, 1.0]	22.0% 24.5% 0.90 [0.73, 1.1]	5.5% 7.6% 0.72 [0.49, 1.1]
On GnRH agonist vs Not On ADT	693 4933	693 693	19.8% 23.3% 0.85 [0.69, 1.1]	22.8% 23.7% 0.96 [0.77, 1.2]	5.6% 7.4% 0.76 [0.50, 1.1]
On GnRH antagonist vs Not On ADT	57 4933	56 56	<20.4%* 22.6% 0.90 [0.43, 1.9]	<22.2%* 28.8% 0.77 [0.39, 1.5]	<18.9%* <18.5%* 1.0 [0.46, 2.2]
On abiraterone vs Not On ADT	182 4933	180 180	10.5% 20.8% 0.50 [0.29, 0.88]	18.2% 22.4% 0.81 [0.51, 1.3]	7.4% 6.4% 1.2 [0.53, 2.6]
On bicalutamide vs Not On ADT	290 4933	288 288	20.8% 22.1% 0.94 [0.68, 1.3]	21.9% 24.0% 0.91 [0.66, 1.3]	6.3% 8.2% 0.77 [0.42, 1.4]
On leuprolide vs Not On ADT	591 4933	590 590	19.3% 23.0% 0.84 [0.67, 1.1]	23.3% 24.2% 0.97 [0.77, 1.2]	6.1% 7.5% 0.82 [0.53, 1.3]

*PSM* propensity score matching, *CI* confidence interval, *ADT* androgen deprivation therapy.

*Denotes ≤10 instances.

Reintervention, complication, and infection rates for patients on GnRH agonists and GnRH antagonists did not significantly differ from those not on ADT. Patients on abiraterone experienced significantly fewer reinterventions (10.5% vs 20.8%, RR = 0.50 [0.29, 0.88]) but similar rates of complications and infections compared to patients off ADT. There were 288 patients on bicalutamide and 590 on leuprolide after PSM (Supplemental Tables 1E, F). Patients on bicalutamide and leuoprolide experienced similar rates of reinterventions, complications, and infections compared to patients not on ADT.

### IPP patients

For IPP, 13,432 patients were identified, 465 (3.5%) of whom were on ADT and 12,967 (96.5%) of whom were off ADT during the specified time period (Table 3). 464 patients were in each group following PSM. Of patients on ADT, 425 were on GnRH agonists, 64 were on GnRH antagonists, and 89 were on abiraterone after PSM (Supplemental Table 2B–D). After PSM, cohorts were largely similar with the exception that ADT patients had a significant positive difference in one of the four codes used for prostatectomy (CPT 55842) ( <2.2%* vs 0%, p < 0.01); however, the TriNetX rounding mechanism makes it impossible to know if this difference is real (Supplementary Table 2A). Patients on ADT had fewer reinterventions (7.2% vs 12%, RR = 0.60 [0.39, 0.92]) and complications (12.7% vs 18.5%, RR = 0.68 [0.49, 0.95]), but a similar rate of infections (2.9% vs 4.5%, RR = 0.63 [0.32, 1.3]) compared to patients not on ADT (Table 4). On Kaplan Meier analysis, median survival was not met for any outcome; however, log-rank test showed greater survival and hazard ratio showed decreased risk for patients on ADT for reintervention (log-rank p = 0.02, HR = 0.61 [0.39, 0.94]) and complication (log-rank p = 0.03, HR = 0.69 [0.48, 0.98]) (Supplemental Fig. 2), but with no significant difference in infections.

**Table 3:** Baseline demographics – inflatable penile prosthesis.

	Inflatable Penile Prosthesis (Total) N = 13,432	Inflatable Penile Prosthesis (On ADT) N = 465	Inflatable Penile Prosthesis (Not On ADT) N = 12,967
**Age (y)**	62.5 +/− 10.2	67 +/− 8.5	62.5 +/− 10.2
**Race**			
Asian	1%	<2%*	1%
American Indian or Alaska Native	<1%	<2%*	<1%
Black or African American	21%	25%	21%
Native Hawaiian or Other Pacific Islander	<1%	0%	<1%
White	58%	63%	57%
Other Race	5%	5%	5%
Unknown Race	14%	4%	15%
**Prostate cancer (ICD C61)**	27%	93%	25%
**Prostatectomy**			
(CPT 55840)	<1%	<2%*	<1%
(CPT 55842)	<1%	<2%*	<1%
(CPT 55845)	1%	4%	1%
(CPT 1014183)	2%	5%	1%
**Personal history of irradiation (ICD Z92.3)**	3%	18%	2%

*ADT* androgen deprivation therapy.

*Denotes ≤10 instances.

**Table 4:** Risk of reintervention, complication, and infection for patients receiving inflatable penile prosthesis.

Comparison	N before PSM	N after PSM	Reintervention (%, Risk Ratio [95% CI])	Complication (%, Risk Ratio [95% CI])	Infection (%, Risk Ratio [95% CI])
On ADT vs Not On ADT	465 12,967	464 464	7.2% 12% 0.60 [0.39, 0.92]	12.7% 18.5% 0.68 [0.49, 0.95]	2.9% 4.5% 0.63 [0.32, 1.3]
On GnRH agonist vs Not On ADT	426 12,967	425 425	7.4% 11.7% 0.63 [0.41, 0.98]	13.2% 18.2% 0.72 [0.52, 1.0]	2.7% 4.5% 0.59 [0.29, 1.2]
On GnRH antagonist vs Not On ADT	65 12,967	64 64	<17.5%* <16.4%* 1.1 [0.48, 2.4]	<20.4%* <16.7%* 1.2 [0.56, 2.7]	<15.9%* <15.9%* 1 [0.45, 2.2]
On abiraterone vs Not On ADT	89 12,967	89 89	<12.2%* 16.7% 0.73 [0.35, 1.6]	<13.0%* 22.9% 0.57 [0.28, 1.1]	<11.6%* <11.5%* 1.0 [0.44, 2.3]
On bicalutamide vs Not On ADT	214 12,967	213 213	<5.2%* 10.8% 0.48 [0.23, 0.99]	9.8% 13.6% 0.73 [0.41, 1.3]	<4.9%* <4.7%* 1.0 [0.44, 2.4]
On leuprolide vs Not On ADT	356 12,967	355 355	7.6% 12.1% 0.63 [0.39, 1.0]	12.2% 19.3% 0.63 [0.49, 0.91]	<2.9%* 4.8% 0.61 [0.28, 1.3]

*PSM* propensity score matching, *CI* confidence interval, *ADT* androgen deprivation therapy.

*Denotes ≤10 instances.

Compared to those not on ADT, GnRH agonist recipients had fewer reinterventions (7.4% vs 11.7%, RR = 0.63 [0.41, 0.98]) but similar rates of complications and infections. Reintervention, complication, and infection rates for patients on GnRH antagonists and abiraterone did not significantly differ from those not on ADT, with this analysis heavily impacted by low sample size and the TriNetX rounding mechanism.

We identified 213 IPP patients receiving bicalutamide and 355 on leuprolide after PSM (Supplemental Table 2E–F). Patients on bicalutamide had significantly fewer reinterventions ( <5.2%* vs 10.8%, RR = 0.48 [0.23, 0.99]), but similar rates of complications and infections than those off ADT. Compared to patients not on ADT, those on leuprolide had fewer complications (12.2% vs 19.3%, RR = 0.63 [0.43, 0.91]), but similar rates of reinterventions and infections.

## Discussion

How ADT impacts AUS and IPP outcomes is, thus far, not well known. Based on our findings, patients on ADT have similar outcomes when receiving an AUS or IPP compared to patients not on ADT. Despite utilizing a generous sample size from a large research database, many of our analysis are still impacted by overall low sample size. Nevertheless, the results should be reassuring for providers looking to place an implant in a patient on ADT.

For AUS, there was almost no difference in outcomes. The only sub-analysis that demonstrated statistical significance was patients on abiraterone had lower rates of reinterventions compared to controls. In fact, abiraterone was associated with about half the rate of reintervention compared to controls with a robust sample size of 180 in each cohort following PSM. Given this combination of sample size and pronounced difference in reintervention, we tend to believe this represents a real finding and not related to bias in our study. The physiologic mechanism behind this finding is not readily apparent but may benefit from further basic science research. Abiraterone is particularly effective in castrate resistant prostate cancer as it targets enzymes involved in androgen biosynthesis which allows it to inhibit extragonadal androgen production as well as gonadal [14]. For this reason, abiraterone is used in later stages of prostate cancer treatment. Perhaps patients on abiraterone have less time to develop an issue requiring or desiring reintervention on their AUS given their advanced cancer and shorter life expectancy.

Regarding IPP, multiple analyses demonstrated lower rates of reinterventions. Specifically, our overall ADT analysis as well as GnRH agonists and bicalutamide. Additionally, our overall ADT analysis and leuoprolide were associated with fewer complications. The exact physiologic mechanism behind these findings is unclear. We hypothesize some of the findings may be related to patient use patterns. In theory, patients on ADT may be more fatigued as a side effect of the medication and have lower libido. These patients may be using their penile implants less and be less motivated to seek reintervention or experience fewer complications given their lack of use.

Our own group previously found that patients with hypogonadism experienced increased rates of reinterventions, complications, and infections following implant surgery [10]. This stands in contrast to this current study demonstrating no significant differences or lower rates or reinterventions or complications for patients on ADT. This may suggest a clinical difference in physiologic hypogonadism compared to pharmacological for patients on ADT. A major limitation of our study is that we are unable to determine the length of time patients were on ADT. Organically hypogonadal patients likely underwent a prolonged decline in androgen levels before displaying overt symptoms and receiving a formal diagnosis. Conversely, patients on ADT experience a rapid decline in androgen levels. Therefore, it is possible that patients with physiologic hypogonadism, on average, spend more time in a hypogonadal state compared to our cohort of patients on ADT and that this may play a significant role in the differences observed. Although other work has shown acute physiologic changes after just one month of androgen deprivation, this work has not focused specifically on penile or urethral tissue health [15]. Additionally, ADT induces extremes in hypogonadism as the goal is to eliminate androgen production. Perhaps, this plays a role in our findings. Further, it is not known how the timing of low androgen levels, as in prior to, during, or after prosthetic implant surgery, factors into the observed findings. Patients who are on ADT at the time of surgery may fare worse compared to patients who did not begin ADT until after the main tissue recovery period, but this was not captured in our study. Alternatively, perhaps the level of androgen deprivation results in patient use or clinical changes that results in this disparity, and it is not physiologically based.

Nonetheless, our findings seem to support those previously reported in the literature. Mann and colleagues found that ADT was not a significant predictor of early AUS device erosion [16]. Bailey and colleagues in a retrospective study with AUS patients only, failed to show any difference in infections, erosions, or urethral atrophy between ADT and ADT-naive patients [17]. Notably, their inclusion criteria specified a minimum of 6 months on ADT within 2 years prior to AUS placement. They note that radical prostatectomy is associated with genital atrophy independent of ADT use and suggest that further atrophy from androgen deprivation may not significantly worsen tissue health compared to non-prostatectomy patients [17].

Major limitations are inherent to this retrospective database study. We are reliant on the data as collected within the TriNetX database which may carry misclassifications. We focused on the initial placement of AUS or IPP, we did not evaluate patients who underwent revision or placement through an infected field as these have a higher risk for complications. We wanted to focus on the initial surgery since this is the main decision point for patients who are considering surgery. We are additionally unable to examine patient charts to determine granular details such as surgeon-specific factors, specific complications, or cause for reintervention. We are unable to determine patient compliance with medications, as well as specific androgen levels, length of time on any medication, and continuous vs intermittent ADT. Further, we cannot generate any causative inferences from our results but use the findings to suggest associations and generate hypotheses.

Limitations notwithstanding, our study has several important clinical implications. Our results suggested a potential benefit, especially regarding reinterventions, with various ADT medications. Even if we think our results may be biased by some of the limitations, it is easy to argue patients suffer no worse outcomes on these medications and potentially even better outcomes. This may be reassuring to providers who know of the impact of hypogonadism on implant outcomes and may be hesitant to offer AUS or IPP to patients receiving ADT. We additionally generated a large sample size for our study, unlikely to be replicated outside of similar database work, which gives strength to our findings and should make our results broadly generalizable. Although the mechanisms behind our results are not readily apparent, the takeaways from the study remain. Ongoing investigation to generate a better understanding of urethral and penile health and the relationship with androgens will be of benefit.

## Conclusion

Many patients with a history of prostate cancer are impacted by bothersome erectile dysfunction or stress urinary incontinence. Thus far, outcomes for patients who take androgen depriving medications for treatment of their prostate cancer who also undergo urologic implant surgery are largely unknown. We found that patients who underwent IPP or AUS surgery do not fare worse when on ADT. We thus conclude that, based on this study, ADT should not limit implant surgery. Further work evaluating the potential impact of duration of ADT therapy and to elucidate mechanisms for these findings is warranted in future efforts.

## Supplementary information

Supplemental Figures

Supplemental Tables

## Data Availability

Data proprietary to TriNetX.
