# Supplementary material for: Androgen deprivation therapy does not increase rates for reintervention, complication, or infection in primary penile implant or artificial urinary sphincter surgery: a retrospective cohort study from the TriNetX network
Source: Int J Impot Res. 2025 Jan 29;37(11):896–901. doi: 10.1038/s41443-025-01015-8 (PMC12623237; doi:10.1038/s41443-025-01015-8)
Supplement: Supplementary file 1 — Supplemental Figures [file 41443_2025_1015_MOESM1_ESM.docx]

**Supplemental Figure 1:** Kaplan Meier survival curve for artificial urinary sphincter patients on androgen deprivation therapy vs not on androgen deprivation therapy for A) reintervention, B) complication, and C) infection.

1A.

Median Survival (days, 4007 vs 5627), log-rank p=0.11, hazard ratio 0.83 [0.66, 1.05]

1B:

Median Survival (not met vs 3722 days), log-rank p=0.18, hazard ratio 0.85 [0.68, 1.08]

1C:

Median Survival (not met), log-rank p=0.16, hazard ratio 0.75 [0.50, 1.13]

**Supplemental Figure 2:** Kaplan Meier survival curve for inflatable penile prosthesis patients on androgen deprivation therapy vs not on androgen deprivation therapy for A) reintervention, B) complication, and C) infection.

2A:

Median Survival (not met), log-rank p=0.02, hazard ratio 0.61 [0.39, 0.94]

2B:

Median Survival (not met), log-rank p=0.03, hazard ratio 0.69 [0.48, 0.98]

2C:

Median Survival (not met), log-rank p=0.19, hazard ratio 0.63 [0.32, 1.26]
