# Supplementary material for: Androgen deprivation therapy does not increase rates for reintervention, complication, or infection in primary penile implant or artificial urinary sphincter surgery: a retrospective cohort study from the TriNetX network
Source: Int J Impot Res. 2025 Jan 29;37(11):896–901. doi: 10.1038/s41443-025-01015-8 (PMC12623237; doi:10.1038/s41443-025-01015-8)
Supplement: Supplementary file 2 — Supplemental Tables [file 41443_2025_1015_MOESM2_ESM.docx]

**Supplemental Table 1**: Cohort characteristics after propensity score matching for artificial urinary sphincter patients A) on ADT vs not on ADT, B) on GnRH agonists vs not on ADT, C) on GnRH antagonist vs not on ADT, D) on abiraterone vs not on ADT, E) on bicalutamide vs not on ADT, and F) on Leuprolide vs not on ADT.

| ***A) On ADT vs Not On ADT*** | | | | | |
| --- | --- | --- | --- | --- | --- |
| Characteristic | Mean +/- SD | Patients (N) | % of Cohort | P-value | Std Diff. |
| Age at index (y) | 70.3 +/- 7.8 vs 70.4 +/- 7.6 | 743 vs 743 | 100% vs 100% | 0.688 | 0.021 |
| Prostate cancer (ICD C61) |  | 696 vs 696 | 93.7% vs 93.7% | 1 | <0.001 |
| Prostatectomy  (CPT 55840)  (CPT 55842)  (CPT 55845)  (CPT 1014183) |  | 10* vs 10*  0 vs 0  33 vs 29  43 vs 34 | <1.3%* vs <1.3%*  0% vs 0%  4.4% vs 3.9%  5.8% vs 4.6% | 1  0.604  0.292 | <0.001  0.027  0.055 |
| Personal history of irradiation (ICD Z92.3) |  | 223 vs 223 | 30.0% vs 30.0% | 1 | <0.001 |
| ***B) On GnRH agonist vs Not On ADT*** | | | | | |
| Characteristic | Mean +/- SD | Patients (N) | % of Cohort | P-value | Std Diff. |
| Age at index (y) | 70.1 +/- 7.8 vs 70.1 +/- 7.8 | 693 vs 693 | 100% vs 100% | 0.940 | 0.004 |
| Prostate cancer (ICD C61) |  | 649 vs 649 | 93.7% vs 93.7% | 1 | <0.001 |
| Prostatectomy  (CPT 55840)  (CPT 55842)  (CPT 55845)  (CPT 1014183) |  | 10* vs 10*  0 vs 0  31 vs 34  40 vs 36 | <1.4%* vs <1.4*  0% vs 0%  4.5% vs 4.9%  5.8% vs 5.2% | 1  0.703  0.637 | <0.001  0.021  0.025 |
| Personal history of irradiation (ICD Z92.3) |  | 205 vs 205 | 29.6% vs 29.6% | 1 | <0.001 |
| ***C) On GnRH antagonist vs Not On ADT*** | | | | | |
| Characteristic | Mean +/- SD | Patients (N) | % of Cohort | P-value | Std Diff. |
| Age at index (y) | 73.3 +/- 6.9 vs 73.6 +/- 7.2 | 56 vs 56 | 100% vs 100% | 0.820 | 0.043 |
| Prostate cancer (ICD C61) |  | 55 vs 55 | 98.2% vs 98.2% | 1 | <0.001 |
| Prostatectomy  (CPT 55840)  (CPT 55842)  (CPT 55845)  (CPT 1014183) |  | 10* vs 0  0 vs 0  10* vs 10*  10* vs 10* | <17.9%* vs 0%  0% vs 0%  <17.9%* vs <17.9%*  <17.9%* vs <17.9%* | <0.001  1  1 | 0.659  <0.001  <0.001 |
| Personal history of irradiation (ICD Z92.3) |  | 16 vs 16 | 28.6% vs 28.6% | 1 | <0.001 |
| ***D) On abiraterone vs Not On ADT*** | | | | | |
| Characteristic | Mean +/- SD | Patients (N) | % of Cohort | P-value | Std Diff. |
| Age at index (y) | 71.3 +/- 7.5 vs 71.1 +/- 7.7 | 180 vs 180 | 100% vs 100% | 0.830 | 0.023 |
| Prostate cancer (ICD C61) |  | 175 vs 175 | 97.2% vs 97.2% | 1 | <0.001 |
| Prostatectomy  (CPT 55840)  (CPT 55842)  (CPT 55845)  (CPT 1014183) |  | 10* vs 10*  0 vs 0  10* vs 10*  11 vs 10* | <5.6%* vs <5.6%*  0% vs 0%  <5.6%* vs <5.6%*  6.1% vs <5.6%* | 1  1  0.822 | <0.001  <0.001  0.024 |
| Personal history of irradiation (ICD Z92.3) |  | 79 vs 80 | 43.9% vs 44.4% | 0.916 | 0.011 |
| ***E) On bicalutamide vs Not On ADT*** | | | | | |
| Characteristic | Mean +/- SD | Patients (N) | % of Cohort | P-value | Std Diff. |
| Age at index (y) | 71 +/- 7.9 vs 71.3 +/- 7.8 | 288 vs 288 | 100% vs 100% | 0.672 | 0.035 |
| Prostate cancer (ICD C61) |  | 274 vs 274 | 95.1% vs 95.1% | 1 | <0.001 |
| Prostatectomy  (CPT 55840)  (CPT 55842)  (CPT 55845)  (CPT 1014183) |  | 10* vs 10*  10* vs 10*  14 vs 15  19 vs 18 | <3.5%* vs <3.5%*  <3.5%* vs <3.5%*  4.9% vs 5.2%  6.6% vs 6.3% | 1  1  0.849  0.865 | <0.001  <0.001  0.016  0.014 |
| Personal history of irradiation (ICD Z92.3) |  | 87 vs 86 | 30.2% vs 29.9% | 0.928 | 0.008 |
| ***F) On Leuprolide vs Not On ADT*** | | | | | |
| Characteristic | Mean +/- SD | Patients (N) | % of Cohort | P-value | Std Diff. |
| Age at index (y) | 70.2 +/- 7.9 vs 70.2 +/- 7.9 | 590 vs 590 | 100% vs 100% | 0.962 | 0.003 |
| Prostate cancer (ICD C61) |  | 559 vs 559 | 94.7% vs 94.7% | 1 | <0.001 |
| Prostatectomy  (CPT 55840)  (CPT 55842)  (CPT 55845)  (CPT 1014183) |  | 10* vs 10*  0 vs 0  29 vs 29  36 vs 31 | <1.7%* vs <1.7%*  0% vs 0%  4.9% vs 4.9%  6.1% vs 5.3% | 1  1  0.529 | <0.001  <0.001  0.037 |
| Personal history of irradiation (ICD Z92.3) |  | 188 vs 188 | 31.9% vs 31.9% | 1 | <0.001 |

ADT (androgen deprivation therapy); SD (standard deviation); Std. Diff. (standardized mean difference)

*Denotes ≤10 instances

**Supplemental Table 2**: Cohort characteristics after propensity score matching for inflatable penile prosthesis patients A) on ADT vs not on ADT, B) on GnRH agonist vs not on ADT, C) on GnRH antagonist vs not on ADT, D) on abiraterone vs not on ADT, E) on bicalutamide vs not on ADT, and F) on Leuprolide vs not on ADT.

| ***A) On ADT vs Not On ADT*** | | | | | |
| --- | --- | --- | --- | --- | --- |
| Characteristic | Mean +/- SD | Patients (N) | % of Cohort | P-value | Std Diff. |
| Age at index (y) | 67 +/- 8.5 vs 67 +/- 8.4 | 464 vs 464 | 100% vs 100% | 0.947 | 0.004 |
| Prostate cancer (ICD C61) |  | 432 vs 432 | 93.1% vs 93.1% | 1 | <0.001 |
| Prostatectomy  (CPT 55840)  (CPT 55842)  (CPT 55845)  (CPT 1014183) |  | 10* vs 10*  10* vs 0  18 vs 12  22 vs 17 | <2.2%* vs <2.2%*  <2.2%* vs 0%  3.9% vs 2.6%  4.7% vs 3.7% | 1  0.002  0.266  0.413 | <0.001  0.210  0.073  0.054 |
| Personal history of irradiation (ICD Z92.3) |  | 82 vs 83 | 17.7% vs 17.9% | 0.932 | 0.006 |
| ***B) On GnRH agonist vs Not On ADT*** | | | | | |
| Characteristic | Mean +/- SD | Patients (N) | % of Cohort | P-value | Std Diff. |
| Age at index (y) | 67.1 +/- 8.6 vs 67.1 +/- 8.5 | 425 vs 425 | 100% vs 100% | .981 | 0.002 |
| Prostate cancer (ICD C61) |  | 394 vs 394 | 92.7% vs 92.7% | 1 | <0.001 |
| Prostatectomy  (CPT 55840)  (CPT 55842)  (CPT 55845)  (CPT 1014183) |  | 10* vs 10*  10* vs 10*  17 vs 11  21 vs 15 | <2.4% vs <2.4%  <2.4% vs <2.4%  4% vs 2.6%  4.9% vs 3.5% | 1  1  0.249  0.307 | <0.001  <0.001  0.079  0.070 |
| Personal history of irradiation (ICD Z92.3) |  | 78 vs 79 | 18.4% vs 18.6% | 0.930 | 0.006 |
| ***C) On GnRH antagonist vs Not On ADT*** | | | | | |
| Characteristic | Mean +/- SD | Patients (N) | % of Cohort | P-value | Std Diff. |
| Age at index (y) | 68 +/- 7.3 vs 68 +/- 7.3 | 64 vs 64 | 100% vs 100% | 1 | <0.001 |
| Prostate cancer (ICD C61) |  | 63 vs 63 | 98.4% vs 98.4% | 1 | <0.001 |
| Prostatectomy  (CPT 55840)  (CPT 55842)  (CPT 55845)  (CPT 1014183) |  | 10* vs 10*  0 vs 0  10* vs 10*  10* vs 10* | <15.6%* vs <15.6%*  0% vs 0%  <15.6%* vs <15.6%*  <15.6%* vs <15.6%* | 1  1  1 | <0.001  <0.001  <0.001 |
| Personal history of irradiation (ICD Z92.3) |  | 13 vs 13 | 20.3% vs 20.3% | 1 | <0.001 |
| ***D) On abiraterone vs Not On ADT*** | | | | | |
| Characteristic | Mean +/- SD | Patients (N) | % of Cohort | P-value | Std Diff. |
| Age at index (y) | 67.1 +/- 7.7 vs 67.1 +/- 7.7 | 89 vs 89 | 100% vs 100% | 1 | <0.001 |
| Prostate cancer (ICD C61) |  | 86 vs 86 | 96.6% vs 96.6% | 1 | <0.001 |
| Prostatectomy  (CPT 55840)  (CPT 55842)  (CPT 55845)  (CPT 1014183) |  | 0 vs 0  0 vs 0  10* vs10*  10* vs 10* | 0% vs 0%  0% vs 0%  <11.2%* vs <11.2%*  <11.2%* vs <11.2%* | 1  1 | <0.001  <0.001 |
| Personal history of irradiation (ICD Z92.3) |  | 29 vs 29 | 32.6% vs 32.6% | 1 | <0.001 |
| ***E) On bicalutamide vs Not On ADT*** | | | | | |
| Characteristic | Mean +/- SD | Patients (N) | % of Cohort | P-value | Std Diff. |
| Age at index (y) | 68.3 +/- 8.9 vs 68.3 +/- 8.8 | 213 vs 213 | 100% vs 100% | 0.956 | 0.005 |
| Prostate cancer (ICD C61) |  | 198 vs 198 | 93.0% vs 93.0% | 1 | <0.001 |
| Prostatectomy  (CPT 55840)  (CPT 55842)  (CPT 55845)  (CPT 1014183) |  | 10* vs 10*  10* vs 0  10* vs 10*  13 vs 11 | <4.7%* vs <4.7%*  <4.7%* vs 0%  <4.7%* vs <4.7%*  6.1% vs 5.2% | 1  0.001  1  0.674 | <0.001  0.314  <0.001  0.041 |
| Personal history of irradiation (ICD Z92.3) |  | 42 vs 42 | 19.7% vs 19.7% | 1 | <0.001 |
| ***F) On Leuprolide vs Not On ADT*** | | | | | |
| Characteristic | Mean +/- SD | Patients (N) | % of Cohort | P-value | Std Diff. |
| Age at index (y) | 66.9 +/- 8.5 vs 66.9 +/- 8.4 | 355 vs 355 | 100% vs 100% | 0.989 | 0.001 |
| Prostate cancer (ICD C61) |  | 336 vs 366 | 94.6% vs 94.6% | 1 | <0.001 |
| Prostatectomy  (CPT 55840)  (CPT 55842)  (CPT 55845)  (CPT 1014183) |  | 10* vs 10*  10* vs 10*  13 vs 10*  17 vs 11 | <2.8%* vs <2.8%*  <2.8%* vs <2.8%*  3.7% vs <2.8%*  4.8% vs 3.1% | 1  1  0.525  0.247 | <0.001  <0.001  0.048  0.087 |
| Personal history of irradiation (ICD Z92.3) |  | 68 vs 69 | 19.2% vs 19.4% | 0.924 | 0.007 |

ADT (androgen deprivation therapy); SD (standard deviation); Std. Diff. (standardized mean difference)

*Denotes ≤10 instances
